# Mogamulizumab-induced drug eruption during multiagent chemotherapy: A possible CD8^+^ T cell-mediated mechanism

**DOI:** 10.1016/j.jdcr.2026.01.014

**Published:** 2026-01-19

**Authors:** Sakiho Inayoshi, Kazunari Sugita

**Affiliations:** Division of Dermatology, Department of Internal Medicine, Faculty of Medicine, Saga University, Saga, Japan

*To the Editor:* We read with interest the insightful comments by Aphale et al, published in December 2025, regarding our previously published article.^1^^,^^2^ As shown in our case, mogamulizumab may be administered during multiagent chemotherapy,^2^ which, as noted in the comment, can complicate the initial immunological landscape, making identification of the causative agent based on skin biopsy alone challenging.^1^

From the existing literature, the clinical and histopathological features of mogamulizumab-associated eruptions during multiagent therapy can be summarized as follows: (1) persistence of cutaneous eruptions despite discontinuation of the suspected culprit agents and (2) interface dermatitis predominantly characterized by CD8^+^ T-cell infiltration.^3^ Reports of mogamulizumab monotherapy have likewise demonstrated CD8^+^ T cell-mediated reactions as characteristic histopathological features.^4^ From a clinical perspective, reports of severe cutaneous adverse reactions, including Stevens-Johnson syndrome and toxic epidermal necrolysis, occurring during mogamulizumab monotherapy are consistent with CD8^+^ T cell-mediated immune responses.^5^^,^^6^

Mechanistically, emerging evidence suggests that, even in mycosis fungoides, mogamulizumab targeting CCR4-expressing CD4^+^ T cells, including regulatory T cells, can lead to sustained activation of CD8^+^ T cells and CXCR3^+^Th1 cells, resulting in a CD8-dominant immune milieu.^7^ Taken together, evidence from previously reported cases indicates that the key immunological features of mogamulizumab-induced reactions include activation of CD8^+^T cells and Th1-skewed immunity, which in turn are associated with characteristic histopathological findings of drug eruption, including predominant CD8^+^ T cell infiltration within lesional skin.

Nonetheless, how these immunological features are modulated in the context of multiagent chemotherapy remains unclear and warrants further investigation through additional clinical and mechanistic studies.

## Conflicts of interest

None disclosed.
